# Chest physiotherapy guided by electrical impedance tomography in high-dependency unit patients with pulmonary diseases: an introduction of methodology and feasibility

**DOI:** 10.1186/s13054-023-04308-w

**Published:** 2023-01-17

**Authors:** Qing Li, Yi Li, Guangyu Niu, Mingna Li, Jia Deng, Knut Möller, Inéz Frerichs, Jianing Xi, Hongying Jiang, Zhanqi Zhao

**Affiliations:** 1 Department of Pulmonary and Critical Care Medicine, Beijing Rehabilitation Hospital, Capital Medical University, Badachu Road, Shijingshan District, 100144 Beijing, China; 2 Institute of Technical Medicine, Furtwangen University, Jakob-Kienzle-Strasse 17, 78054 Villingen-Schwenningen, Germany; 3 Department of Anaesthesiology and Intensive Care Medicine, University Medical Centre of Schleswig-Holstein Campus Kiel, Kiel, Germany

**To the Editor**

Chest physiotherapy (CPT) has been widely used as an adjunctive treatment to facilitate airway clearance and promote effective coughing [1]. Previous studies indicated that CPT could improve lung function and prevent ventilator-associated pneumonia [2]. One main challenging issue with current CPT practice is that the objective assessment of ventilation distribution is missing [3]. Consequently, the CPT plan is not fully personalized according to individual needs.

Electrical impedance tomography (EIT) is a radiation-free functional imaging technology that monitors ventilation and perfusion at bedside [4]. EIT could potentially provide feedback during CPT to allow its personalized adaptation to individual needs. However, no study has investigated the feasibility to guide CPT using EIT so far.

The aim of the prospective study was to introduce the EIT-guided CPT methodology developed in our center, to analyze its feasibility in patients with airway secretion retention and clearance impairment and to describe feedback from the end-users, both patients and physiotherapists, on its use.

Patients with airway secretion retention and clearance impairment are evaluated for the eligibility of EIT-guided CPT. Untreated pneumothorax and moderate-to-large pleural effusion are contraindications for CPT. In addition, patients with contraindications for EIT measurement are not eligible.

Individualized EIT-guided CPT program is developed for eligible patients. The program consists of 3 parts: (1) understanding of patient’s respiratory status through spatial and temporal ventilation distribution; (2) developing individualized CPT plans accordingly; and (3) evaluating the efficacy and adjusting CPT where necessary. The workflow is illustrated in Fig. 1. Detailed adjustments of CPT according to the EIT results are listed in Additional file 1.

**Fig. 1:**
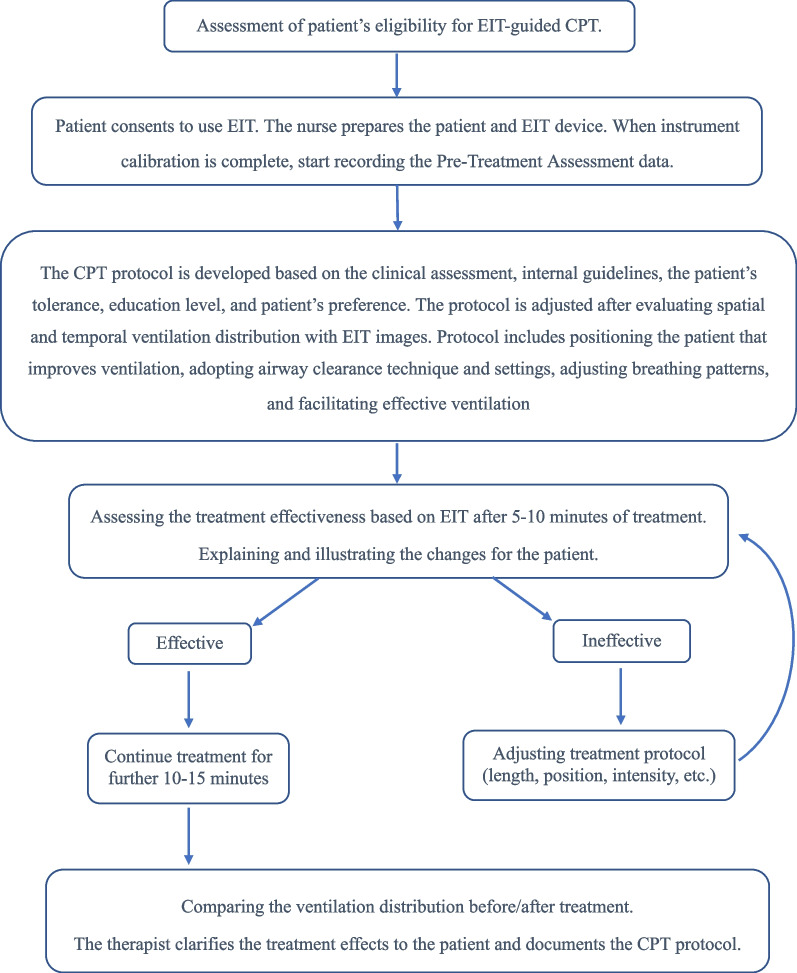
Illustration of workflow for individualized EIT-guided CPT program development

This study was approved by the Ethical committee of Beijing Rehabilitation Hospital (2019bkky-067). Patients with airway secretion retention and clearance impairment admitted to the department from 01.01 to 31.06.2020 were screened. Patient’s inclusion details and outcome assessments are summarized in Additional file 1.

A total of 154 patients were screened for the given period, and 88 were eligible and included. Six patients did not complete the treatment, which corresponded to a dropout rate of 6.8%. Three of them complained about the discomfort of electrode belt application and refused to continue wearing it. The other three patients were not able to cooperate, and the EIT data quality was not good enough for real-time assessment. No adverse events occurred throughout the study.

Finally, 82 patients (93.2%) were able to complete the treatment (58:24, males vs. females; age 69.4 ± 10.7 years; BMI 22.1 ± 1.4 kg/m^2^; APACHE II 16.24 ± 3.52). Eleven patients had pneumonia without comorbidities and 71 with comorbidities, including coronary heart disease (*n* = 18), hypertension (*n* = 43), diabetes (*n* = 22), cerebrovascular events (*n* = 29), malignant tumors (*n* = 12) and renal insufficiency (*n* = 3). Patients were satisfied with the EIT-guided CPT program (satisfaction score 23.82 ± 0.98 out of 30 points). Significant improvement in ventilation distribution was observed in all patients after the entire EIT-guided CPT program (*p* < 0.01). Additional clinical data are summarized in Additional file 1. The program required additional planning and implementation time of 5–10 min. One trained physiotherapist was required to interpret the EIT results and adjust the therapy program accordingly. The training time for an EIT-inexperienced physiotherapist was half-day. Two physiotherapists participated in the study and found that EIT results were helpful and provided accurate and solid evidence to guide CPT.

To our best knowledge, the pilot study demonstrated systematically for the first time the feasibility of a bedside feedback method to guide CPT in patients with airway secretion retention and clearance impairment. Most of the patients were able to complete the CPT program. They were motivated and satisfied with the treatment via the real-time visual feedback.

The most important step of the program is to understand the cause of current respiratory status. The spatial and temporal ventilation distribution of the patients is often heterogeneous. After starting the EIT measurement, poorly ventilated areas are identified through the ventilation distribution (e.g., defect score [5]). The potential causes of poorly ventilated areas are discussed with the physicians combining other clinical evidence. The CPT program is designed or adjusted accordingly. Meanwhile, images are also used to illustrate changes in lung ventilation to inspire the patients. The points of view from physiotherapists and patients are summarized in Additional file 1.

The study has a few limitations. This was a single-center study. Patients who were unconscious and unable to complete the satisfaction assessment were not included. Experience with EIT-guided CPT is limited and could vary among physiotherapists. Since the study objective was to introduce the methodology and examine the feasibility, only a retrospective control group was included (Additional file 1). The effectiveness of the therapy should be verified by comparing with traditional CPT in a randomized-controlled setting. Nevertheless, based on our internal statistic for previous years, the patient dropout rate of CPT program was 11.0% which was much higher than 6.8% in the present study.

## Supplementary Information

**Additional file 1**. Further details of Methods and Discussions.

## Data Availability

Data are available upon reasonable request to the corresponding author.
