# Supplementary material for: Chest physiotherapy guided by electrical impedance tomography in high-dependency unit patients with pulmonary diseases: an introduction of methodology and feasibility
Source: Crit Care. 2023 Jan 17;27:24. doi: 10.1186/s13054-023-04308-w (PMC9847064; doi:10.1186/s13054-023-04308-w)
Supplement: Supplementary file 1 — Additional file 1. Further details of Methods and Discussions. [file 13054_2023_4308_MOESM1_ESM.docx]

Online Supplement

**Chest physiotherapy guided by electrical impedance tomography in high-dependency unit patients with pulmonary diseases: an introduction of methodology and feasibility**

Qing Li^1#^, Yi Li^1#^, Guangyu Niu^1^, Mingna Li^1^, Jia Deng^1^, Knut Möller^2^, Inéz Frerichs^3^, Jianing Xi^1^, Hongying Jiang^1*^, Zhanqi Zhao^2*^

1. Department of Pulmonary and Critical Care Medicine, Beijing Rehabilitation Hospital, Capital Medical University, China

2. Institute of Technical Medicine, Furtwangen University, Villingen-Schwenningen, Germany

3. Department of Anaesthesiology and Intensive Care Medicine, University Medical Centre of Schleswig-Holstein Campus Kiel, Germany

# Authors contributed equally to this work

* Corresponding authors

Dr. Hongying Jiang, Department of Pulmonary and Critical Care Medicine, Beijing Rehabilitation Hospital, Capital Medical University, Xixiazhuang, Badachu Road, Shijingshan District, Beijing 100144, China

Email address: 6jhy@163.com

Dr. Zhanqi Zhao, Institute of Technical Medicine, Furtwangen University, Jakob-Kienzle-Strasse 17, 78054 Villingen-Schwenningen, Germany

Email address: zhanqi.zhao@hs-furtwangen.de

**Methods**

*EIT measurements and EIT-guided strategies*

Two sessions of CPT (morning and afternoon, 20 minutes each) guided by the real-time ventilation distribution imaging obtained with EIT were conducted using a newly developed enhanced 2-week CPT program. The CPT session consisted of modified postural drainage [[1](#_ENREF_1)], assisted cough technique [[2](#_ENREF_2)], positive expiratory pressure [[3](#_ENREF_3)], high-frequency chest wall oscillation [[4](#_ENREF_4)], chest percussion, vibration [[5](#_ENREF_5)], and active cycle of breathing techniques (ACBT) [[6](#_ENREF_6)]. The appointed therapist performed pulmonary auscultation and thoracic palpation to assess the status of pulmonary ventilation and secretion retention, and whether the patient's cough ability could complete effective airway clearance. Individualized program was formed according to the assessment, internal guidelines, the patient's tolerance, education level, and patient’s preference. EIT measurements were conducted using the PulmoVista 500 EIT device (Dräger Medical, Germany) at a scan rate of 20 images/s. An elastic belt with 16 electrodes was placed at the patient's chest wall between the 4^th^ and 5^th^ intercostal space and a reference electrode at the middle of the patient's abdomen. Continuous EIT scanning was performed during the whole CPT session and the generated images were used to guide the CPT treatments.

*EIT-guided modified postural drainage combined with vibrations and chest percussion*: tidal variation images in EIT reveals heterogeneously ventilated regions. Physiotherapist identified such regions at the bedside and instructed the patient to take the appropriate drainage position, so that the poorly ventilated regions became gravity non-dependent regions. Subsequently, the physiotherapist put her hands on the poorly ventilated area with a vibratory force. A compressive pressure was produced by the therapist’s arms.


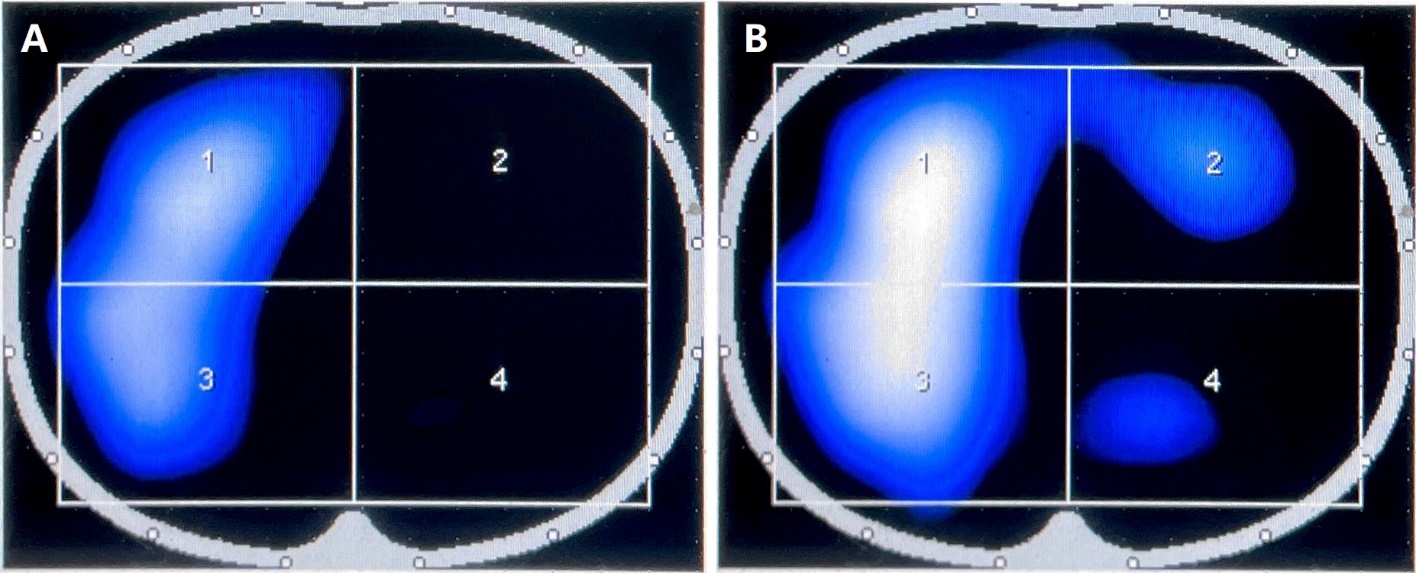


S-Figure 1. Effective modified postural drainage after identifying the ventilation defect regions (left lung in the present case). A: Pre-guiding ventilation distribution; B: Post-guiding ventilation distribution (with right lateral position).

*EIT-guided ACBT*: ACBT is one of the most commonly used airway clearance techniques. It consists of repeated cycles of three ventilatory phases: breathing control, thoracic expansion, and forced expiration technique (FET). EIT was used to guide thoracic expansion and FET. ① EIT-guided thoracic expansion: EIT displays dynamically the inhalation and exhalation of air in real time. When instructing the patient to perform thoracic expansion, the physiotherapist shared the real-time images with the patient and guided the patient to expand the thorax based on visual feedback to improve ventilation in poorly ventilated areas. ② EIT-guided FET: FET involves one or two huffs. One is a slow huff from the middle lung volume down to a low lung volume. The other one is a quicker huff that requires patient to quickly exhale from a high lung volume to a low lung volume. By comparing the former with the latter lung volume change using the impedance-time curve, the physiotherapist was able to assess whether the patient's inspiratory volume reached the middle lung volume and high lung volume.


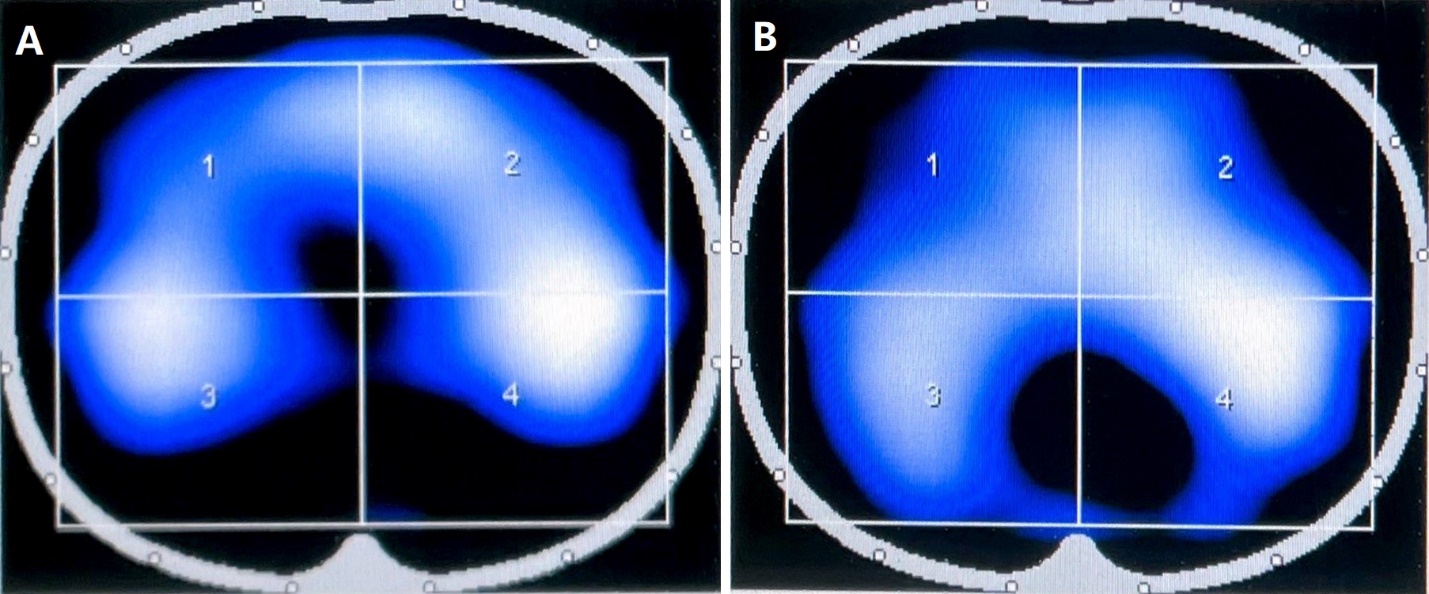


S-Figure 2. Effective ACBT treatment after promoting sputum mobilization. A: Pre-guiding ventilation distribution; B: Post-guiding ventilation distribution.

*EIT-guided effective coughing*: Adequate inspiratory volumes for a cough should be at least 60% of the vital capacity for that individual [[7](#_ENREF_7)]. Similar to EIT-guided FET, the physiotherapist was able to assess whether the patient reached the adequate inspiratory volume for effective coughing.


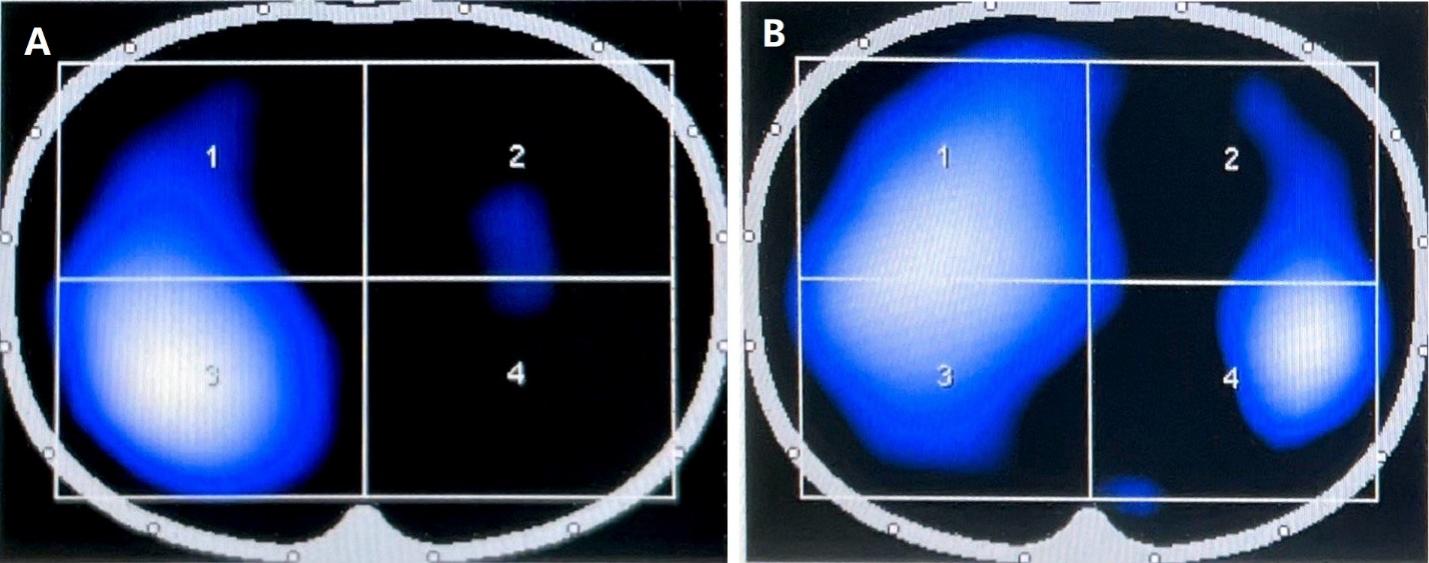


S-Figure 3. EIT-guided effective coughing. A: Pre-guiding ineffective cough (inhale phase); B: Post-guiding effective cough (inhale phase).

*EIT as a tool for instant feedback and motivation*: After each treatment session, the effect of the treatment was assessed immediately. The ventilation improvement was visible and explained using the EIT images. The patient visualized the improvement and was motivated for further CPT sessions.


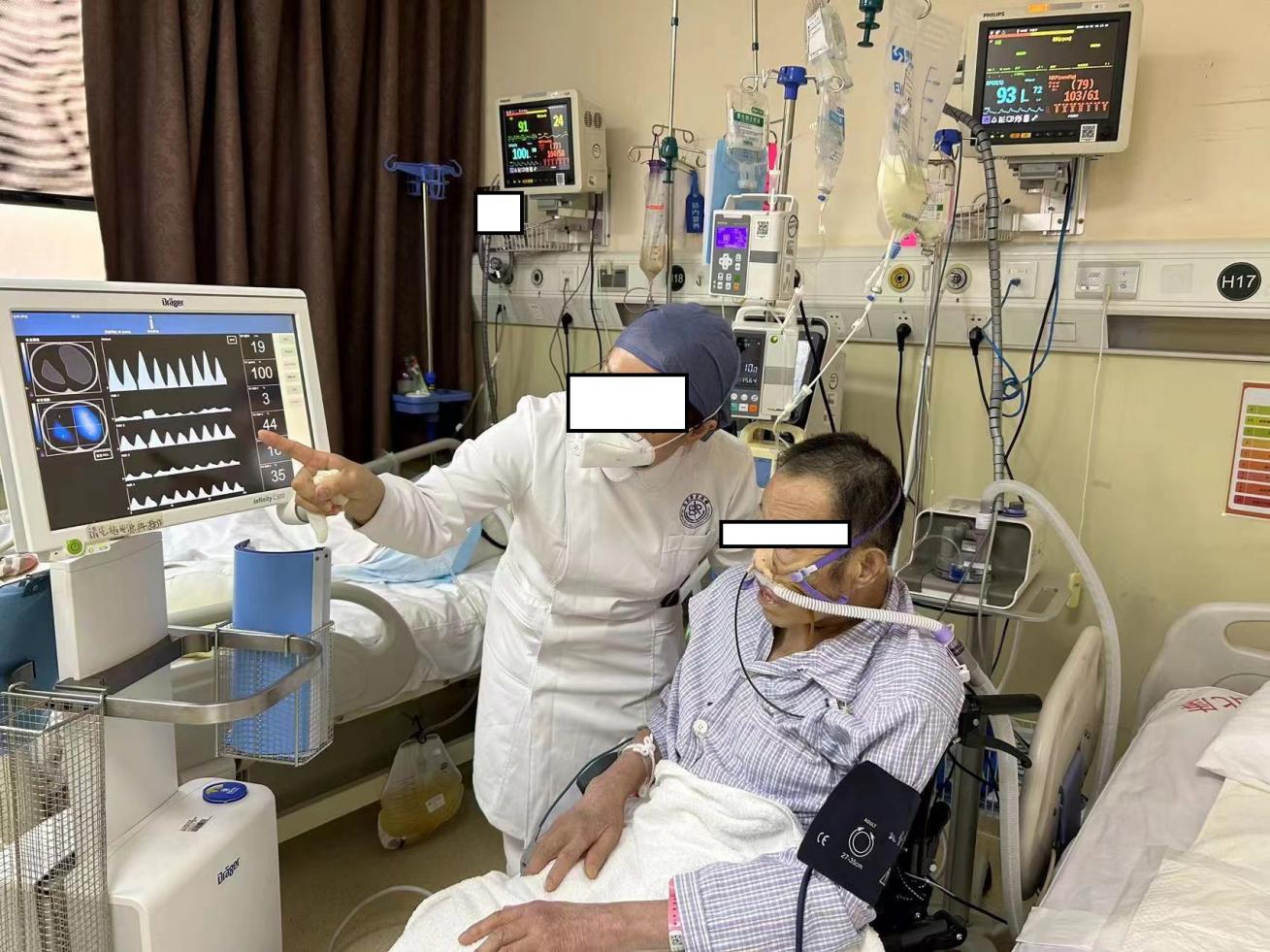


S-Figure 4. EIT images as visual feedback for a patient having CPT.

*Patients*

The study was conducted in a rehabilitation hospital. In our department (Pulmonary and Critical Care Medicine), patients are critically ill with clinically relatively stable situation but still have single or multisystem organ failure. The current study included patients who have left the ICU but cannot be transferred to the general ward (so-called “high-dependency care units”, HDU patients) [8].

Patients meeting the eligibility criteria described in the previous section were enrolled. The exclusion criteria were 1. viral pneumonia; 2. thoracic spinal deformity; 3. pegnant or lactating women; 4. inability to cooperate with pulmonary rehabilitation; 5. acute exacerbation of chronic obstructive pulmonary disease or acute attack of bronchial asthma. All participants provided written informed consent.

To demonstrate the clinical benefit of the EIT-guided program, patients treated with traditional program (without EIT) from July 1 to December 31, 2019 were retrospectively reviewed as control group. Clinical Pulmonary Infection Score (CPIS), length of stay and cost of hospitalization were evaluated.

*Outcome measures and data analysis*

The primary outcome was the acceptability of the patients, including the dropout rate and patients’ satisfaction with the treatment. Secondary outcomes included acceptability of physiotherapists, and practicalities of the program (e.g. additional workload, adverse events, preparation time for EIT measurement and physiotherapists’ education). Exploratory outcomes were the difference in CPIS, length of stay and cost between the prospective study group and the retrospective control group. Patient’s satisfaction was assessed by a survey questionnaire administered at the end of the treatment phase. The questionnaire was a 5-point Likert-scaled response satisfaction survey. The questionnaire from the Australian Lung Foundation pulmonary rehabilitation toolkit was adopted and modified [[9](#_ENREF_9)]. The six-item measure asks the participants to rate the effectiveness of the CPT components on a scale of 1 = strongly disagree to 5 = strongly agree (S-Table 1). Cronbach’s alpha of the scale was 0.86. The item regarding physical exercise program was excluded.

S-Table 1. Satisfaction survey

| *Please tick the relevant column for your answer to each statement below:* | Strongly agree  5 | Agree 4 | Neutral 3 | Disagree 2 | Strongly disagree 1 |
| --- | --- | --- | --- | --- | --- |
| The program has helped me manage my lung condition more effectively |  |  |  |  |  |
| The information was pitched at an appropriate level |  |  |  |  |  |
| Overall the program met my expectations |  |  |  |  |  |
| I found the program was worthwhile. |  |  |  |  |  |
| I would recommend this program to others with a lung condition |  |  |  |  |  |

Scores range from 1 = strongly disagree to 5 = strongly agree.

**Results**

A total of 82 patients were included in this treatment, among which 7 were mechanically ventilated, 65 were on high-flow oxygen therapy, and 10 were non-invasive ventilation. Chest x-ray indicated improvement after the treatment in 57 subjects and the findings matched that from EIT measurements. Tidal volume before and after treatment were available for the patients under invasive ventilation (n=7) and the changes were statistically significant (S-Table 2). Respiratory rate and oxygen saturation (SpO_2_) at 30% oxygen concentration were recorded for all studied subjects. Statistically significant improvements were found after the treatment. These results are summarized in S-Table 2.

S-Table 2. Comparison of tidal volume, respiratory rate and oxygen saturation before and after treatment

|  | n | before | after | t value | P value |
| --- | --- | --- | --- | --- | --- |
| Tidal volume (ml) | 7 | 333.80±65.91 | 436.20±28.90 | 3.596 | 0.023 |
| Respiratory rate (breathe/minute) | 82 | 19.51±3.72 | 16.28±2.66 | 12.039 | <0.001 |
| Oxygen saturation (%) | 82 | 94.85±1.61 | 98.87±1.22 | 29.248 | <0.001 |

A total 79 patients treated with traditional program were retrospectively reviewed. The differences in CPIS between the study group and the control group were insignificant before (T1) and at the end of the treatment (T4), but they became significant on Day 3 and 7 after the treatment start (T2 and T3; S-Table 3). The patients in both study and control group would only be discharged after the symptoms have been improved. Hence, the CPIS score was similar at T4. Nevertheless, the length of stay was significantly shorter and the cost of hospitalization was significantly lower in the study group (S-Table 4).

S-Table 3. Comparison of CPIS between two groups of patients at different time-points

| Time-points | Study group (n=82) | Control group (n=79) | t value | p value |
| --- | --- | --- | --- | --- |
| T1 | 8.9±0.57 | 8.66±0.73 | 2.718 | 0.093 |
| T2 | 5.42±0.67 | 7.29±0.91 | 8.673 | 0.004 |
| T3 | 2.79±0.71 | 3.68±0.67 | 5.139 | 0.025 |
| T4 | 0.54±0.51 | 0.66±0.53 | 0.025 | 0.889 |
| p value | <0.0001 | <0.0001 |  |  |

T1, before treatment (Day 0); T2 and T3, Day 3 and Day 7 after the treatment started; T4, at the end of treatment before discharge.

S-Table 4. Comparison of length of stay and cost of hospitalization between the two groups

|  | Study group (n=82) | Control group (n=79) | t/Z value | p value |
| --- | --- | --- | --- | --- |
| Length of stay (day) | 8.91±1.11 | 10.29±1.78 | 7.434 | 0.006 |
| Cost of hospitalization (CNY) | 18729.40±2381.45 | 23191.40±2939.61 | 7.226 | 0.007 |

**Discussion**

*Physiotherapist’s point of view*

Based on the positive results of this study, the EIT-guided CPT became a routine training program for our physiotherapists in the department. With a half-day training time, colleagues are able to manage the use of EIT to guide the therapy. Taking the assessment of secretion retention in the lungs for example, with airway clearance therapy such as thorax expansion and assisted cough technique, a certain amount of airway secretions can be drained. When the CPT is performed without the guidance of EIT, auscultation is conducted to confirm the effectiveness. The disappearance of moist rales in a lung lobe imply that the secretions have been cleared. However, if the moist rales are still heard in another lobe, it is unknown whether this is caused by secretion retention. If EIT is available and poor ventilation is noted in the lung lobe with persisting rales, further airway clearance therapy will focus on this lung region. Nevertheless, due to the limited parameters available in the online software of the EIT device, the interpretation of the patient status according to the images and the development of the corresponding treatment plans were intuitive but still subjective. In the future, standardized data analysis and treatment strategies should be developed and validated.

*Patients’ point of view*

The advantages of CPT are not well advertised. Based on the internal statistic for previous years, the patient dropout rate of CPT program was 11.0% which was higher than 6.8% in the present study. Some patients are reluctant to cooperate with the physiotherapists during the treatment program because they do not think CPT can help them. The patients might not be able to feel the improvement within the first couple of sessions. With the intuitive visualization and real-time feedback from the EIT images, the patients can better understand the rationale of the treatment, as well as the improvement in regional ventilation distribution. The cooperation and compliance of the patients during the treatment sessions are increased with the help of EIT, which can be observed in daily practice and measured through patient-reported outcomes. Hereby the feedback from a patient who received EIT-guided CPT was quoted: “I knew how to work on my lung during breath training. I understood my lung condition and why I need to receive CPT to improve the breathing pattern and cough.”

**References**

1. Sultanpuram S, Alaparthi GK, Krishnakumar SK, Ottayil ZC. **Physiotherapy Practice Patterns for Management of Patients Undergoing Thoracic Surgeries in India: A Survey**. *Surg Res Pract.* 2016;2016:9717489.

2. Spinou A: **A Review on Cough Augmentation Techniques: Assisted Inspiration, Assisted Expiration and Their Combination**. *Physiol Res* 2020, **69**(Suppl 1):S93-S103.

3. McIlwaine M, Button B, Nevitt SJ: **Positive expiratory pressure physiotherapy for airway clearance in people with cystic fibrosis**. *Cochrane Database Syst Rev* 2019, **2019**(11).

4. Longhini F, Bruni A, Garofalo E, Ronco C, Gusmano A, Cammarota G, Pasin L, Frigerio P, Chiumello D, Navalesi P: **Chest physiotherapy improves lung aeration in hypersecretive critically ill patients: a pilot randomized physiological study**. *Critical Care* 2020, **24**(1):479.

5. Wilson LM, Morrison L, Robinson KA: **Airway clearance techniques for cystic fibrosis: an overview of Cochrane systematic reviews**. *Cochrane Database Syst Rev* 2019, **1**:CD011231.

6. Uzmezoglu B, Altiay G, Ozdemir L, Tuna H, Sut N: **The Efficacy of Flutter((R)) and Active Cycle of Breathing Techniques in Patients with Bronchiectasis: A Prospective, Randomized, Comparative Study**. *Turk Thorac J* 2018, **19**(3):103-109.

7. Frownfelter D, Dean E: **Cardiovascular and Pulmonary Physical Therapy: Evidence to Practice**, 5th edn: Elsevier; 2012.

8. Ohbe H, Sasabuchi Y, Yamana H etc. **Intensive care unit versus high-dependency care unit for mechanically ventilated patients with pneumonia**: a nationwide comparative effectiveness study. Lancet Reg Health West Pac. 2021 Jul 5;13:100185.

9. **The Pulmonary Rehabilitation Toolkit** [<http://www.pulmonaryrehab.com.au>] accessed on July 1, 2021
